# A Simulation Study Using Terrestrial LiDAR Point Cloud Data to Quantify Spectral Variability of a Broad-Leaved Forest Canopy

**DOI:** 10.3390/s18103357

**Published:** 2018-10-08

**Authors:** Renato Cifuentes, Dimitry Van der Zande, Christian Salas-Eljatib, Jamshid Farifteh, Pol Coppin

**Affiliations:** 1 Hémera Centro de Observación de la Tierra, Facultad de Ciencias, Universidad Mayor, Santiago 8340589, Chile; 2 Directorate Natural Environment, Royal Belgian Institute of Natural Sciences, 1000 Brussels, Belgium; dimitry.vanderzande@naturalsciences.be; 3 Centro de Modelación y Monitoreo de Ecosistemas, Facultad de Ciencias, Universidad Mayor, Santiago 8340589, Chile; cseljatib@gmail.com; 4 Laboratorio de Biometría, Universidad de La Frontera, Temuco 4811230, Chile; 5 Department of Biosystem, Katholieke Universiteit Leuven, 3000 Leuven, Belgium; farifteh@alumni.itc.nl (J.F.); pol.coppin@kuleuven.be (P.C.)

**Keywords:** canopy structure, leaf area density, leaf area index, ray tracing, PBRT, vegetation index

## Abstract

In this analysis, a method for construction of forest canopy three-dimensional (3D) models from terrestrial LiDAR was used for assessing the influence of structural changes on reflectance for an even-aged forest in Belgium. The necessary data were extracted by the developed method, as well as it was registered the adjacent point-clouds, and the canopy elements were classified. Based on a voxelized approach, leaf area index (LAI) and the vertical distribution of leaf area density (LAD) of the forest canopy were derived. Canopy–radiation interactions were simulated in a ray tracing environment, giving suitable illumination properties and optical attributes of the different canopy elements. Canopy structure was modified in terms of LAI and LAD for hyperspectral measurements. It was found that the effect of a 10% increase in LAI on NIR reflectance can be equal to change caused by translating 50% of leaf area from top to lower layers. As presented, changes in structure did affect vegetation indices associated with LAI and chlorophyll content. Overall, the work demonstrated the ability of terrestrial LiDAR for detailed canopy assessments and revealed the high complexity of the relationship between vertical LAD and reflectance.

## 1. Introduction

Remote sensing technologies are extensively used in forestry for mapping physical-structural features of the land and in forest surveys. Monitoring forest health and stress from remotely sensed images is a priority for forest management. There are several applications of optical remote sensing in assessing biophysical and physiological characteristics of forest ecosystems in order to estimate and predict forest ecosystem health and sustainability [1].

Hyperspectral (HS) remote sensing data retrieved from space and airborne sensors, such as Hyperion (http://eo1.usgs.gov/sensors/hyperion) and APEX (www.apex-esa.org), respectively, can deliver information on e.g., forest biochemical composition, that is essential for research on nutrient cycling, vegetation stress, biomass, and species composition among others [2]. Some examples of these studies of biophysical analysis using HS remote sensing data include tree species classification [3], deriving leaf area index (LAI), estimating chlorophyll content [4], and assessing forest biomass [5].

The spectral response of forest canopies has been proved to be affected by internal factors, such as canopy geometry, height, vigor, and species composition [6]. Canopy geometry (i.e., canopy closure and density) has been documented by Guyot et al. [7] as the most significant factor in the optical properties of forest canopies. All these factors should be taken into account when using HS remote sensing from forested areas. The influence of canopy structure on solar radiation interception and transmittance can be quantified using three-dimensional (3D) radiative transfer models [8]. The majority of solar radiation models in plant canopies are based on the radiative transfer concept, where one of the central questions is how to describe the forest environment in terms of the 3D distribution of the area volume density of their foliage elements. A fundamental input for developing such models encompass a correct description of canopy structure in terms of tree position, crown shapes, leaf density, and spatial distribution of leaf area [9]. This heterogeneity of canopy structure is also greatly influenced by the clumping index and the leaf inclination angle distribution [10,11]. The leaf inclination angle is defined as the angle between the leaf surface normal and the zenith. Difficulties in accessing forest or tree canopies plus the difficulties in applying actual methods using consistent and replicable protocols, impose severe limitations for direct sampling and quantification of most of the above-mentioned variables. Therefore, an accurate representation of the spatial variation of the canopy structure, both vertical and horizontal, is hard to achieve.

Applications of light detection and ranging (LiDAR) technology for large-scale forest mapping using airborne platforms, also known as airborne laser scanning (ALS), have satisfactorily produced tree crown and forest canopy level characterizations as detailed in Leiterer et al. [12], contributed to the study of ecosystems by fusing 3D data with spectral data [13], and provided thorough measurements for accurate representation of vertical and horizontal dimensions of vegetation canopies [14,15,16]. However, various ALS-based studies have applied assumptions of diversified tree crown archetypes [17,18]. Terrestrial laser scanning (TLS) has demonstrated great capabilities to represent forest environments at different degrees of complexity [19,20]. Depending on the characteristics of the LiDAR instrument, TLS provides an important opportunity for 3D modeling of forest canopies for simulations of the internal canopy radiation regime through radiative transfer models. Modeling approaches have predominantly focused on using TLS point cloud datasets, employing several assumptions on growth patterns and tree foliage characteristics [21].

Canopy structure has been represented and evaluated by models assuming homogeneous green plant material and optical thickness given by LAI, as in Broge and Leblanc [22], but also multi-layer (vertical gradients of leaf optical properties) and multi-element one-dimensional models have been proposed to describe vertically heterogeneous canopies [23,24]. Moreover, simulation of other effects—such as the hot-spot and horizontal discontinuity of canopies—have been included in extensions or combinations of the SAIL (scattered by arbitrary inclined leaves) reflectance model with geometric models [25]. For forested environments, additional variables such as crown cover and tree shape need to be considered to make the model suitable for analysis [26]. 3D forest canopy models from LiDAR data offer a comprehensive representation of trees, delivering accurate information on canopy structure and composition, and could be a relevant input for the development of radiative transfer models. The effect of the vertical distribution of leaf area (the third dimension) on the HS response of forest canopies retrieved from space or airborne sensors as described in this study, is presented and analyzed to highlight the importance of counting with reference and realistic data of the vertical heterogeneity of canopies.

This study used TLS data and a novel methodology for reconstruction of a real forest canopy in 3D, allowing to quantify LAI and LAD. Applying Monte Carlo ray tracing techniques, the spectral variability derived by changes in these structural measures was explored. The physically-based ray tracer (PBRT, [27]) was used to model light-surface interactions by creating a forest scene with particular canopy geometry descriptions, material optical properties, the source of illumination and sensor platform, among other components. The spectral variability was ultimately expressed as percentage change on the estimation of both chlorophyll and LAI related vegetation indices.

## 2. Materials and Methods

The canopy model was built using 3D data (henceforward called point cloud) collected by a phased-based terrestrial LiDAR scanner (TLS) FARO^®^ LS880 (FARO^®^ Technologies Inc., Stuttgart, Germany) using a continuous-wave laser at 785 nm (76 m maximum range) on a pure beech (*Fagus sylvatica* L.) forest stand in Heverlee Forest (Flanders, Belgium). The average stand variables are: 223 trees/ha, basal area of 28.6 m^2^/ha, 40.4 cm diameter at breast height (DBH). Average tree height of 32.3 m, and height to crown base of 14.3 m were derived from the point cloud.

**Figure 1:**
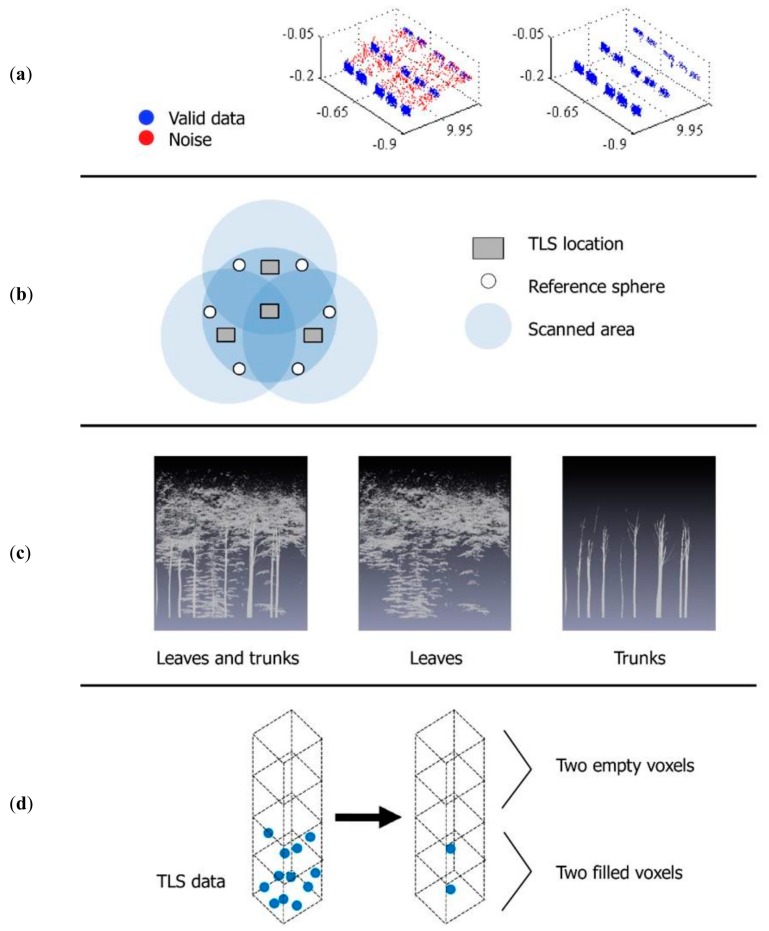
Processing steps for TLS data: (**a**) Noise filtering: noise (red dots) is removed from point clouds; (**b**) Registration: point clouds generated from scanned areas (top view) are merged in a common coordinate system using reference spheres; (**c**) Classification: point clouds (grey dots) are classified into leaves and trunks; (**d**) Voxelization: 3D space is divided into a finite number of cubes or voxels (black segmented line) which are given attributes (empty, filled) depending on the interaction between the laser beam and the voxel.

The complete point cloud processing consists of four steps: ghost point filtering, registration, classification, and voxelization. A distance-based filter was applied to the scan points re-projected in a 2D format (Figure 1a), where each point was examined and removed if predefined allocation and distance criteria relative to its neighborhood (a kernel box) were not fulfilled, as in Cifuentes et al. [28]. Their study on correcting ghost points was conducted under controlled conditions and evidenced that there are a number of variables influencing ghost points occurrence, including that the sensitivity of the algorithm decreases with range. Taking this into account, plus forest stand conditions (e.g., density and tree height) providing our ranges of interest, a rather intensive filtering was applied as follows: a scan point was recognized as valid if the difference in distance between the point being evaluated and the rest of the points in the kernel was smaller than a distance threshold of 0.02 m. The allocation threshold is the percentage of scan points in the kernel that falls within the distance threshold, and it was set to 75%. It is important to note that possible over filtering can be resolved by using other techniques, namely voxelization, which is explained in a subsequent paragraph. Reference spheres were used to register nine point clouds into one comprehensive point cloud dataset (Figure 1b), partly overcoming both TLS limitation in range and occlusion effect.

Classification of point clouds into leaves and trunks was done in two steps (Figure 1c): (i) subdividing and connecting the points from a point cloud using octrees [29]; and (ii) classifying trunks as the sets of sub-clouds where the dimension of their bounding boxes fulfilled specific requirements: the length of the bounding box in height (*z*-axis) was at least three times the length of the bounding box on the shortest horizontal axis (*x* or *y*), and the ratio between these two horizontal axes (*x* and *y*) was between 0.66 and 1.5, in order to capture vertically-oriented elements. In this way, we selected sub-clouds where points were arranged, at least, with an assumed branch angle of 150°. According to Bayer et al. [30], pure beech forest stand can have branch angles as high as 140°. Remaining sub-clouds were classified as leaves. In this work, we adapted the voxel-based approach for 3D tree modeling used by Van der Zande et al. [31] for assessing light environment variability in forest canopies. The 3D space was divided into a finite number of cubic voxels with a voxel side length of 2 cm, following recommendations from literature for this particular forest stand [32], and they were classified depending on the interaction between the scan point and the voxel itself. Voxels with at least one return in it were assigned a value of 1 (filled); a value 0 (empty) was given to voxels that did not enclose any return (Figure 1d).

The physically-based ray tracer (PBRT, [27]) is a Monte Carlo rendering system that supports the implementation of different models for light-surface interaction, sensor types, and the illumination source. An approach adapted from Stuckens et al. [33] was used in this work as follows: (i) Trunks were built as triangular meshes by applying the ball-pivoting algorithm described by Bernardini et al. [34] and leaf voxels were abstracted by triangles with a fixed area of 400 mm², based on the approach introduced by Van der Zande et al. [31] who used larger discs instead of triangles to characterize leaves of *Quercus robur* L., and supported by the findings of Cifuentes et al. [32] where triangular leaves of variable area (100–900 mm^2^) were tested depending on voxel side length; (ii) the leaf inclination angle defined as the angle between the leaf surface normal and the zenith was not measured in the field. In the present study, it was assumed for each leaf to have a fixed angle of 42.5°. This fixed value matched the average zenith angle of beech leaves of simulated trees for 3D modeling of light interception, as presented in Van der Zande et al. [11]. This value has been adopted given that in natural beech forest stands the plagiophile leaf angle distribution seems more suited to estimate canopy structural attributes and to model radiation transmission when no reference leaf inclination angle data are available [35,36]. The azimuth of the leaves, in turn, was set randomly between 0° and 360°; (iii) a homogeneous forested area of 150 × 150 m was created adding 24 instances (or clones) of the 30 × 30 m core area; (iv) For tree leaves, reflectance spectra were measured during the field campaign using a FieldSpec^®^ 3 plant probe and leaf clip with the black background panel to calculate the average diffuse reflectance and transmittance spectra [32,37], and a bidirectional scattering distribution function (BSDF) model was employed [38]. For trunks, one measured spectrum and a Lambertian reflectance model was defined [33]; (v) LAI was calculated from the canopy model built using the LiDAR data.

**Figure 2:**
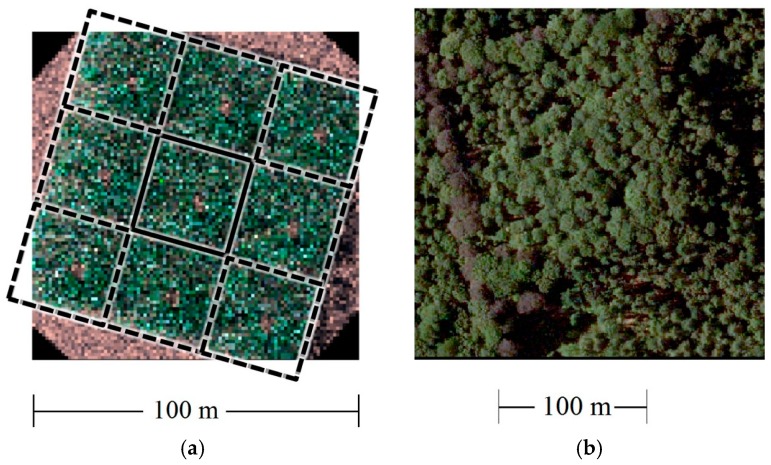
(**a**) Raytraced image (RGB) of the simulated forest in PBRT. The core area (black solid line) in the center, with eight neighboring cloned areas (black segmented line), can be seen; (**b**) An image from the real forest is also displayed. Source orthophoto: Informatie Vlaanderen.

LAI variability in natural environments is driven by weather conditions, season, and species competition for light, among other factors [39,40]. Similarly, the effects of disturbances caused by natural processes or management can shape the vertical distribution of LAD. After, four canopy structure configurations were analyzed: (i) the configuration with reference LAI and LAD (LR); (ii) the configuration with reduced LAI by 5% (L5); (iii) the configuration with reduced LAI by 10% (L10); and (iv) the configuration with reference LAI but modified LAD profile (LRT) by translating 50% of leaf area from the upper part (≥17 m) to lower parts of the 3D space (<17 m and >1.3 m). To simulate the scene illumination a directional light source for the direct (unscattered) light and a skymap that contains the angular distribution of diffuse light were used. Direct and diffuse illuminations were calculated from 350–2500 nm with a 10 nm interval. A measured soil spectrum with no spatial variability was used as soil background. Finally, the light transport algorithm was implemented by an integrator that computed reflected radiance from surfaces in the scene. Elements of the scene can be visualized in Figure 2.

In order to simulate an HS device to measure top-of-canopy reflectance, a sensor with a spectral range from 400 to 2500 nm and spectral resolution of 10 nm was specified in PBRT. The hemispherical-directional reflectance is then retrieved by means of a sensor with orthographic projection placed over the simulated canopy, with a spot size of 21 × 21 m. Thus, simulations were restricted to the 21 × 21 m central area to avoid errors due to lateral radiation fluxes.

Two different categories of vegetation indices were calculated using the reflectance information retrieved from the simulated forest canopy: chlorophyll content related, and LAI related indices (Table 1).

## 3. Results

**Figure 3:**
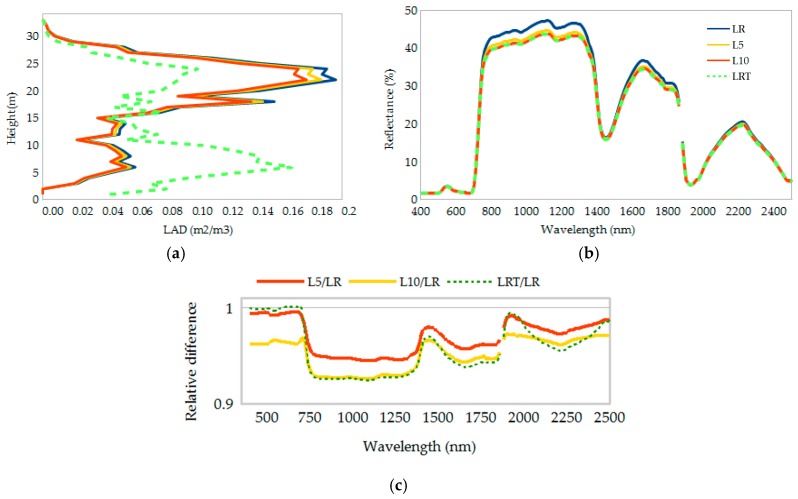
(**a**) Vertical profiles of leaf area density (LAD) for the simulated forest canopy with reference leaf area index (LR: 2.32 m^2^/m^2^), LR reduced by 5% and 10% (L5: 2.22 m^2^/m^2^ and L10: 2.09 m^2^/m^2^, respectively), and LR with modified LAD distribution (LRT: 2.32 m^2^/m^2^); Measured spectra from simulated canopies expressed as reflectance (**b**) and relative difference to LR (**c**).

The calculated LAI for the modeled canopy was 2.32 m^2^/m^2^. Beech forest stands in Flanders, Belgium, with similar DBH, basal area, and tree density, have delivered an average LAI assessed by hemispherical photography of 2.88 m^2^/m^2^ [39]. The difference can be attributable to the approach used (i.e., passive camera versus active laser) and the occlusion effect since TLS coverage at higher parts of dense canopies is limited, hence forest canopy biomass is underestimated. The graphical representation of the changes in LAI and in the vertical distribution of LAD are shown in Figure 3a. The calculated spectral response from top-of-canopy and its variability after changes in structure is also presented (Figure 3b). When these changes occur, differences in the spectral response of forest canopies are notorious in the near- and shortwave-infrared regions (NIR and SWIR, respectively).

The reflectance of L5 and L10 are almost identical. Nevertheless, reducing LAI by 5% and 10%, resulted in an average decrease in reflectance in the NIR region of 5.4% and 7.2%, respectively (Figure 3b). These results confirm the positive relationship between NIR reflectance and LAI. The magnitude of the reduction was also influenced by a decrease in shadow, which intensified NIR reflectance. Differences become more clear throughout the spectral range (350–2500 nm) in Figure 3c. The LRT curve almost overlaps L5 (visible), while clearly overlaps L10 (NIR and part of SWIR). The reduction in LAI as a percentage (5% or 10%) at each height level, means that the number of removed leaves is greater at higher parts in the canopy (20–25 m), given this particular vertical profile of LAD. For the LRT canopy, the higher relative differences in reflectance are also in NIR (average 7.3%), followed by the SWIR and visible regions (Figure 3c). Changes in the visible range (i.e., 400–700 nm) were, in this case, the lower ones (<0.3%) from the three different canopy structure configurations. The high similarity between the response from the L10 canopy and the LRT canopy is evident on the 750–1300 nm and 1700–1900 nm spectral range. The LRT configuration may be seen (or assumed) as a slightly more open canopy than the original scenario, with LAD concentrated on juvenile broad-leaved trees. Calculated canopy openness was 18.7% from simulated hemispherical photography of the modeled canopy. The overall reflectance in this complex canopy will be dominated by NIR reflectance from canopy layers more exposed to shadows (closer to the ground), with suppressed NIR reflectance due to their presence, making the spectral response of LRT almost identical to the one from L10.

**Figure 4:**
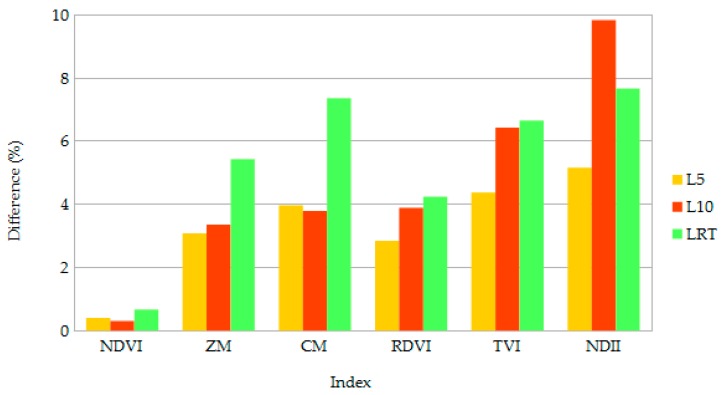
Percentage difference of calculated vegetation indices for three different canopy configurations. Leaf area index reduced by 5% and 10% (L5 and L10, respectively), and LR with modified LAD distribution (LRT).

The presented changes in reflectance, especially in the NIR region, transferred clear effects to calculated vegetation indices, as presented in Figure 4. The NDVI was the less influenced index when altering canopy structure, with change <0.7%. Here, even though the LAI was not modified in LRT, this canopy configuration showed the higher relative difference. A similar situation was observed in the chlorophyll content related indices ZM and CM, with 5.4% and 7.4% relative difference, respectively, and for the LAI related indices RDVI and TVI, with 4.2% and 7.2% relative difference, respectively. Finally, L10 displayed the higher relative difference for the NDII (9.8%), followed by LRT (7.7%).

The effect of background (e.g., ground surface, rocks, litter materials) in areas with low canopy cover added to the calculated influence of canopy structure, can produce a range of vegetation index values since clumping increases the contribution of the soil background by increasing the gap fraction [33]. Spectral mixing methods have been used to model the relative contributions of background elements to spectral response. Results obtained in the present simulation study may complement the extensive work and publications coming from the community working on canopy modeling and remote sensing measurement inversion techniques, to better understand the influence of vertical canopy structure variations on the image spectra.

## 4. Discussion

The similarities between LRT reflectance and L10 reflectance are emphasized, as the aim of the present work is to highlight that there is a need to quantify and understand the effect of the vertical LAD profile in the spectral response of forest canopies. This is clearly represented in Figure 3c, where it is possible to appreciate that the ratio L10 to LR and the ratio LRT to LR (except in the visible) appear almost identical. These effects can have a significant impact on several scientific applications, for example in canopy cover prediction incorporating NIR reflectance [46], or recognition and classification of trees from hyperspectral imagery using visible and NIR reflectance [47].

For the LRT canopy in Figure 4, chlorophyll content and LAI related vegetation indices exhibit inconsistencies that can be deducted from the variation in the NIR part of the spectrum. Is it important to note here that even though the NDII is a normalized index same as the NDVI, it shows high sensitivity to changes in canopy structure. It has been highlighted in early research that there is a high correlation between the ratio of the middle-infrared band to the NIR band, and LAI [48,49]. The NDII, also named normalized difference water index (NDWI) in some studies [50] has been widely used to estimate equivalent water thickness of leaves and canopies, and has been linked and correlated to LAI. Considering that leaf reflectance in the SWIR part of the electromagnetic spectrum is dominated by liquid water absorption, it has been assumed that the NDII is correlated to the LAI by integrating individual leaf equivalent water thickness for each leaf layer to have an estimation of the total canopy equivalent water thickness [51]. Furthermore, the impact on calculated vegetation indices originated from different vertical LAD profiles can have significant effects on several scientific applications, such as on validating protocols for PAR-based LAI measurements in forest environments [52], predicting LAI on crop canopies [53], monitoring fuel moisture content [50], and estimating aboveground biomass [54].

Despite difficulties in gathering leaf inclination angle distribution data due to logistic costs and high variability depending on a number of factors, a pertinent work from Raabe et al. [10] contributed with a suitable approach to characterize and analyse them. Incorporating important variables such as this one would contribute to generating more realistic canopy models. Attention must be paid, however, in assuming spherical leaf angle distribution for tree species from temperate and boreal regions, as denoted by Pisek et al. [36].

Limitations of TLS to characterize tree tops need to be overcome using complementary ALS data. The combination of high spatial resolution and developments of 3D techniques has increased the ability to obtain vegetation information from LiDAR data to characterize canopies and monitor forest status and change [55]. Although ALS is not able to reconstruct forest canopy structure as TLS does, it can unquestionably reveal coarse features such as the penetrations at different crown depths, and the spatial pattern of laser points, among other related characteristics [16].

## 5. Conclusions

A 3D canopy structure of an even-aged forest stand was represented in detail by means of TLS data, through an objective processing chain. Based on simulations performed within a ray tracing environment, the present study investigated the changes in reflectance of forest canopies derived from modifications in LAI and in the vertical distribution of LAD. Under these controlled conditions, the NIR region was more sensitive to changes in LAI, followed by the SWIR and visible parts of the spectrum. Forest canopy with a different distribution of LAD in the vertical plane (LRT) delivered comparable variation in reflectance to the L10 configuration in the NIR part, and to the L5 configuration in the visible part. Variability in both chlorophyll content and LAI related vegetation indices can be deducted from the variation in the NIR part of the spectrum, for the LRT canopy configuration. 

The present communication aimed to draw the attention to the effect of forest canopy structural changes over widely known and used indices, without necessarily meaning more (or less) leaves per tree, but forest canopies with different vertical LAD profiles. The effects of different vertical LAD profiles showed an impact on vegetation indices that can have significant impacts on numerous scientific applications.

The actual restrictions of TLS to represent the upper part of the canopy, that may lead to underestimating forest canopy biomass, may be fixed by using complementary ALS data. As recommended in the literature, this approach has been suitable to characterize and monitor forest canopies at different scales.

As part of a future analysis, it is foreseen to develop new forest scenes and carry out simulations considering a number of other variables, such as the leaf angle distribution, canopy cover, and photosynthetically-active radiation (PAR). Hence, the simulation study presented in this work will possibly become a suitable modelling approach if reflectance and structural measurements are performed and evaluated under similar natural scenarios. In addition, new advances in laser scanning technology will allow further use of TLS to accurately rebuild forest canopies and explore the effects of vertical LAD on different forest ecosystems, in order to model its contribution to the canopy spectral response.

## Figures and Tables

**Table 1:** Formulation of the two categories of biophysical (B) and structural (S) vegetation indices used in this study. R_λ_ is the reflectance at the wavelength λ.

Index	Formulation	Category ^1^
Normalized difference vegetation index (NDVI), [41]	(R_800_ − R_670_)/(R_800_ + R_670_)	B
Zarco and Miller index (ZM), [42]	R_750_/R_710_	B
Carter and Miller (CM), [43]	R_695_/R_760_	B
Renormalized difference vegetation index (RDVI), [44]	(R_800_ − R_670_)/(R_800_ + R_670_)^1/2^	S
Triangular vegetation index (TVI), [22]	0.5 × ((120 × (R_750_ − R_550_) − 200 × (R_670_ + R_550_))	S
Normalized difference infrared index (NDII), [45]	(R_850_ − R_1650_)/(R_850_ + R_1650_)	S

^1^ Two categories are shown. B = biophysical vegetation index; S = structural vegetation index.
